# Comparison of Epidemiology, Clinical Features, and Outcomes of Patients with Reported Ewing Sarcoma and PNET over 40 Years Justifies Current WHO Classification and Treatment Approaches

**DOI:** 10.1155/2018/1712964

**Published:** 2018-08-08

**Authors:** Kevin Campbell, David Shulman, Katherine A. Janeway, Steven G. DuBois

**Affiliations:** Dana-Farber/Boston Children's Cancer and Blood Disorders Center, Harvard Medical School, Boston, MA, USA

## Abstract

**Background:**

As of 2013, the WHO has classified peripheral primitive neuroectodermal tumors (PNETs) within the umbrella of Ewing sarcoma family of tumors (ESFTs) given their shared biology. Histologic features differ between PNET and Ewing sarcoma (ES), and potential clinical differences between PNET and ES have not been fully elucidated.

**Methods:**

Through the National Cancer Institute's Surveillance, Epidemiology, and End Results (SEER) database, we identified 3,575 patients identified with histologic diagnosis of ES or PNET from 1973 to 2014. We used Fisher's exact tests to compare patient and tumor characteristics between groups. Kaplan–Meier methods were used to estimate overall survival.

**Results:**

Patients with ES were more likely to be male, ≤18 years old at diagnosis, white, and hispanic compared to patients with PNET (*p*=0.016 for sex; *p* < 0.001 for all other variables). Patients with PNET were more likely to have soft tissue primary tumors (*p* < 0.001), and among those with bone tumors, a lower rate of axial or pelvic tumors (*p* < 0.001). Patients with PNET had significantly worse 5-year survival compared to ES patients, though the absolute difference was small (51.3% versus 55.5%; *p* < 0.001). Survival of patients with PNET diagnosed in the 1990s or later more closely approximated patients with ES, while patients with PNET diagnosed in the 1980's and earlier had inferior outcomes.

**Conclusions:**

Despite shared underlying biology, patients with PNET and ES show differences in clinical presentation and overall survival, with the latter differences largely mitigated in more recent decades.

## 1. Introduction

Ewing sarcoma family of tumors (ESFTs) encompass Ewing sarcoma (ES) and primitive neuroectodermal tumors (PNETs). ES and PNET are recognized to have a common biology with characteristic recurrent translocations, most commonly *EWSR1/FLI1* [1]. The distinction between these entities is based only on their degree of differentiation. PNETs, despite their name, are slightly less primitive than Ewing tumors and show features of neural differentiation. The 2013 update to the World Health Organization pathology classification system removed any distinction between PNET and ES, though the diagnosis of PNET is still rendered by some pathologists [2]. Given their shared biology, common treatment strategies and clinical trial protocols are now used to treat patients with these tumors.

Several studies from the 1980s and 1990s compared tumor characteristics and outcomes of ES and PNET tumors. While some groups reported that PNET histology carried a worse prognosis than ES [3, 4], other studies observed no difference in clinical outcomes between PNET and ES [5–7]. The average cohort size examined in these studies was 59, with the largest consisting of 120 patients. Importantly, methods distinguishing PNET from ES were not uniformly followed in all these studies [5]. In parallel, multidisciplinary treatment approaches for ES tested in cooperative group clinical trials have led to incremental improvements in outcomes, but only more recent trials have included patients with PNET [8–11].

In the context of an updated WHO pathology classification system, we sought to examine potential differences in patients with ES versus PNET histology using the largest dataset available. Our objectives included an evaluation of patient demographic features and presenting clinical features between groups. We also investigated potential differences in survival in these two groups. As treatment strategies for PNET have shifted to align with ES treatment strategies over the past four decades, we also evaluated changes in outcomes over time.

## 2. Patients and Methods

### 2.1. Patients

We obtained National Cancer Institute's Surveillance, Epidemiology, and End Results (SEER) data from all eighteen registries for the diagnosis years of 1973–2014 using SEER∗Stat version 8.3.4 [12]. These parameters allow for inclusion of approximately 27% of the United States population representing ethnically and geographically diverse areas of the country. Diagnosis of tumors was based on the International Classification of Disease for Oncology, third revision (ICD-O-3) histology codes for ES (code 9260) and PNET (code 9364). To ensure inclusion of only ES or PNET tumors, codes for Askin tumors (code 9365; *n*=15) and CNS PNET (code 9473; *n*=1621) were excluded. We further excluded ES or PNET tumors with primary sites (defined via SEER code ‘Site recode ICD-0-3/WHO 2008') in the CNS (*n*=70) to eliminate potential CNS PNET erroneously coded as peripheral PNET.

### 2.2. Variables

The primary predictor variable was histology coded as ES or PNET based upon ICD-O-3 codes. In this registry-based study, histology was not centrally reviewed and tumor translocation status was not available. Clinical variables included patient demographics and presenting clinical features of the disease, including tumor size (dichotomized at 8 cm), grade (undifferentiated versus any degree of differentiation), stage (distant metastasis versus no distant metastasis), and primary site. Primary site was coded according to ICD-0 code [12]. Primary site was dichotomized as bone versus nonbone given previous literature noting differences in outcomes based upon this distinction [13]. Among primary tumors of the bone, subanalyses were conducted to evaluate frequency of axial versus appendicular location of these tumors. A substantial proportion of patients had missing data for stage, tumor size, and tumor grade.

The sole measure of clinical outcome was overall survival. Overall survival was calculated as time from initial diagnosis to death, with surviving patients censored at time of last follow-up. The median follow-up time for the analyzed cohort was 110 months.

### 2.3. Statistical Analyses

Fisher's exact tests were used to compare categorical variables between groups defined by histology (ES versus PNET). Overall survival was calculated using Kaplan–Meier methods and compared between groups using log-rank tests. All *p* values are two-sided, and *p* < 0.05 was considered statistically significant. All statistical analyses were performed using STATA version 13.

## 3. Results

### 3.1. Patient Demographics Differ between ES and PNET

**Figure 1:**
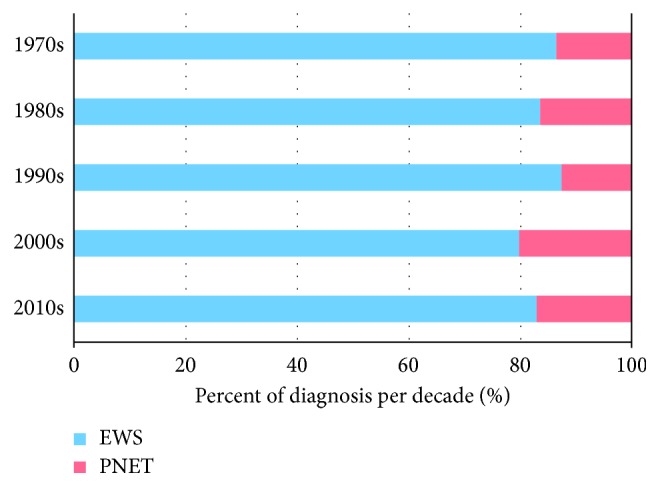
Bar graph depicting proportion of PNET versus ES by decade from 1973 to 2014.

The analytical cohort included 3,575 patients. Of these, 2,945 (82.4%) cases had ES and the remaining 630 (17.6%) cases had PNET. We observed significant differences in several demographic features between PNET and ES (Table 1). Patients with ES were more likely to be male, ≤18 years old at diagnosis, white, and hispanic compared to patients with PNET (*p*=0.016 for sex; *p* < 0.001 for all other variables). Similar findings were seen when focused exclusively on patients diagnosed from 1990 to 2014 (Supplementary Table (available here)). An increased frequency in the diagnosis of PNET as designated by SEER is noted in recent decades, with data indicating 76% of PNET diagnosis occurring from 2000 to 2014 (24% from 1973 to 1999). This pattern is significant compared with ES diagnosis of 68% and 32% from these same time periods (*p* < 0.001; Figure 1).

### 3.2. Presenting Clinical Features Differ between ES and PNET

Table 2 compares presenting clinical features between PNET and ES. There was no difference in rate of metastasis at diagnosis or tumor size between PNET and ES. Tumor grade likewise did not differ between groups, though data were missing for the majority of patients. Patients with ES were more likely to have a bone primary tumor compared to patients with PNET (*p* < 0.001). Subanalyses of patients with bone primary tumors showed that ES patients had significantly higher rates of axial and pelvic sites than PNETs (*p* < 0.001). Similar findings were seen when focused exclusively on patients diagnosed from 1990 to 2014 (Supplemental Table).

### 3.3. Overall Survival Differs between ES and PNET

**Figure 2:**
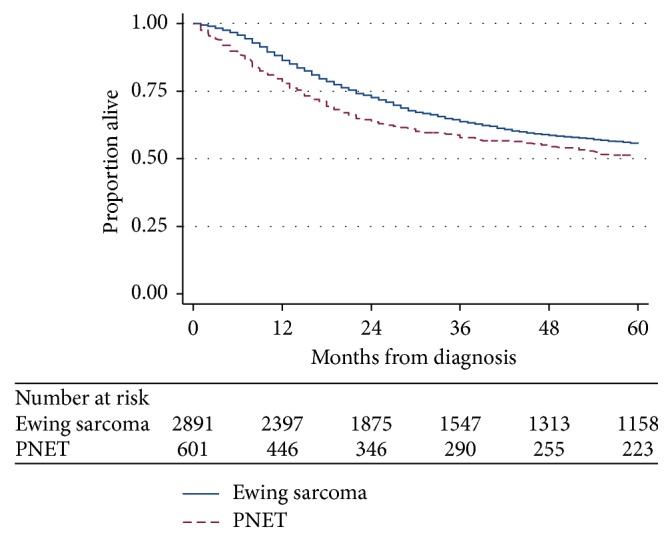
Kaplan–Meier estimates of overall survival from time of diagnosis for patients with Ewing sarcoma or PNET from 1973 to 2014 (*p* value < 0.001).

Overall survival for all patients with ES and PNET is shown in Figure 2. Three- and five-year Kaplan–Meier estimates of overall survival for ES compared to PNET were 63.8% (95% CI 61.9–65.8%) versus 57.8% (95% CI 53.6–61.8%) and 55.5% (95% CI 53.6–57.5%) versus 51.3% (95% CI 47.0–55.5%), respectively (*p* < 0.001).

**Figure 3:**
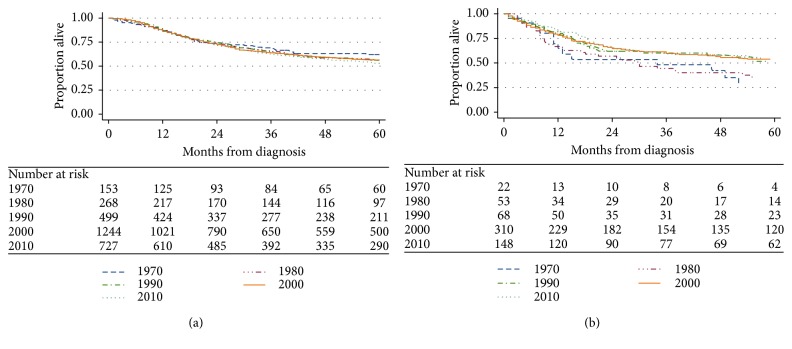
Kaplan–Meier estimates of overall survival from time of diagnosis by decade of diagnosis for patients with (a) Ewing sarcoma (*p* value = 0.35) and (b) PNET (*p* value = 0.08).

**Figure 4:**
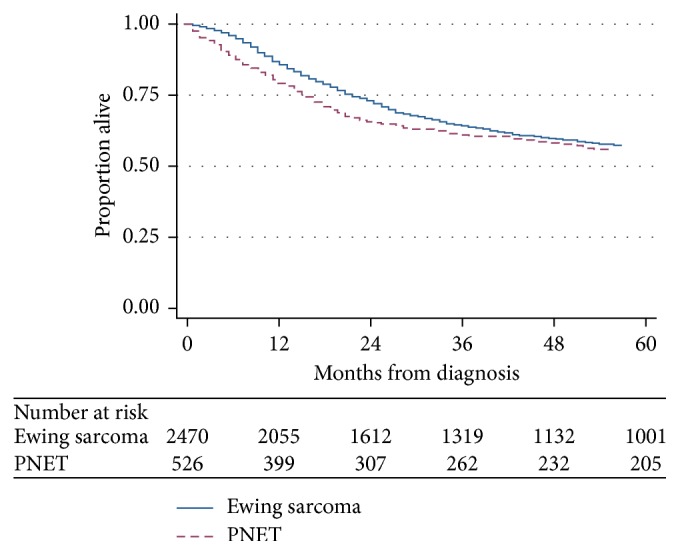
Kaplan–Meier estimates of overall survival from time of diagnosis for patients with Ewing sarcoma or PNET diagnosed in 1990 or later (*p* value = 0.03).

To evaluate this survival difference further, we constructed separate Kaplan–Meier estimates for overall survival according to decade of diagnosis (Figures 3(a) and 3(b)) and also focused exclusively on patients diagnosed in the 1990s and later. Stratified by decade, outcomes for ES did not significantly change (Figure 3(a)), while 5-year OS estimates for PNET were lower in the 1970s and 1980s (28.1% and 35.3%) compared to subsequent decades with OS estimates (all >50%) (Figure 3(b); *p*=0.08). Restricting survival analysis to patients diagnosed in the 1990s and later (Figure 4) narrowed the difference in OS between PNET and ES, though patients with PNET still showed a small, yet statistically significant, survival disadvantage compared to ES (*p*=0.03).

## 4. Discussion

The ESFTs represent a group of cancers that share many common features, most notably at a genetic level. It is not known why some of these tumors that harbor identical translocations nevertheless display histologic differences. Our analysis provides new insight into how these histologic differences translate into differences in presenting features, but only subtle differences in clinical outcomes. Our finding of similar outcomes provides support for the recent WHO decision to combine PNET and ES into one pathologic entity. Of the demographic and clinical differences observed, it is most notable that patients with PNET were older and more likely to have soft tissue primary tumors. We did observe that PNET histology imparts a small, yet statistically significant survival disadvantage versus ES, a finding that may be influenced by varied diagnostic criteria for PNET as well as inadequate outcomes prior to the 1990s. Overall, our analysis provides a large, comprehensive evaluation of clinical differences based upon histologic designation as ES versus PNET.

Several demographic and clinical differences between PNET and ES merit additional discussion. Trends for the epidemiological presentation of ES were largely confirmed, while data regarding sex, race, and age at diagnosis for patients with PNET represent novel findings. Bone as primary site occurred more often (70.1% versus 56.2%) in ES. As well, primary tumors of the bone occurring in areas associated with worse prognosis, the axial skeleton [14] and pelvis [13], occurred significantly more often in ES than PNET. These differences provide new insight into how histologic distinctions between ES and PNET may render meaningful clinical differences. While some of these demographic and clinical features may impact prognosis, the incidence of the strongest adverse clinical prognostic factor in this disease, metastasis at diagnosis, was similar between ES and PNET.

As noted, our finding that patients with PNET had inferior outcomes compared to patients with ES was unexpected but does not oppose the recent integration of the two entities into one pathologic category. PNETs are enriched for soft tissue primary sites, a location previously reported to confer favorable outcome [10, 14, 15]. Our subanalyses based upon decade of diagnosis demonstrate that this survival difference was driven in part by inferior outcomes in patients with PNET treated prior to the 1990s. While unable to prove with SEER data, we postulate that better outcomes in PNET in more recent decades may be due to the adoption of ES treatment strategies and clinical trial protocols for use in PNET patients that, prior to this decade, may have been inappropriately treated with inferior regimens. As evidence, the paramount discovery of increased overall survival after addition of ifosfamide and etoposide for patients with localized ES was also demonstrated for patients with PNET [16]. Indeed, our subanalysis focused exclusively on patients treated in the 1990s and later showed that the difference in outcomes between PNET and ES was largely attenuated. These results corroborate the current uniform treatment approach regardless of PNET or ES histology.

The major strength of our report is the number of patients evaluated. Use of the SEER database allowed for analysis of the largest cohorts of ES and PNET patients available. The SEER database reflects many areas of the United States and is representative of the general population. Our study is novel in our use of population-based data to investigate differences between ES and PNET, though there are several important limitations to our study in the use of SEER data. Foremost, we were unable to centrally review our primary predictor variable, PNET versus ES histology, though the similar proportion of patients with PNET histology in our cohort compared with previously reported single-institution studies with central review [7, 17] is reassuring. As diagnostic criteria for PNET were more heterogeneous in the 1970s and 1980s, we completed a subanalysis focused on patients diagnosed in subsequent decades when diagnostic criteria were more uniform [3]. The results of this analysis help to strengthen our confidence in our overall findings using the full cohort. Data on translocation status are not available in SEER, and therefore, it is not known to what extent patients with PNET harbored typical translocations or potentially may have included some patients with more recently recognized Ewing-like sarcoma entities (e.g., *CIC/DUX4* or *BCOR/CCNB3* tumors) [18, 19]. Our analysis was also limited by incomplete data for large percentages of our subjects regarding several clinical features, including stage, tumor size, and grade. We lack data on the treatment strategies for PNETs during this time period, though past reports indicate some PNET patients were treated on neuroblastoma or rhabdomyosarcoma protocols [3].

In summary, our work supports the current paradigm that groups PNET and ES pathologically and also treats patients with PNET and ES uniformly. We elucidated differences between PNET and ES that extend beyond their histologic differences. However, despite their histologic divergence, patients with PNET treated in a more contemporary era have only a modest survival disadvantage compared to patients with ES. This observation coincides with the timeline during which treatment approaches have become aligned for these two entities.

## Figures and Tables

**Table 1:** Demographic features of patients with Ewing sarcoma and PNET (*n*=3575).

	Ewing sarcoma (*N* = 2945)	PNET (*N* = 630)	*p* value
*N*	*N* (%)	*N* (%)	
Sex			
Male	1768 (60.0)	345 (54.8)	0.016
Female	1177 (40.0)	285 (45.2)	

Age at diagnosis			
0–18 years	1669 (56.7)	235 (37.3)	<0.001
19+ years	1276 (43.3)	395 (62.7)	

Race			
White	2632 (89.7)	532 (85.0)	<0.001
Black	102 (3.5)	43 (6.9)	
Others^*∗*^	199 (6.8)	51 (8.2)	
Unknown		4	

Ethnicity			
Hispanic	604 (20.5)	68 (10.8)	<0.001
Nonhispanic	2341 (79.5)	562 (89.2)	

^*∗*^Asian or Pacific Islander, American Indian, or Alaska native.

**Table 2:** Clinical presenting features of patients with Ewing sarcoma and PNET (*n*=3575).

	Ewing sarcoma (*N *=* *2945)	PNET (*N *= 630)	*p* value
	*N* %	*N* %	
Metastasis at diagnosis			
Yes	323 (24.7)	74 (24.4)	0.941
No	984 (75.3)	229 (75.6)	
Unknown	1638	327	

Maximum tumor dimension			
<8 cm	225 (51.2)	137 (51.7)	0.891
≥8 cm	493 (48.8)	128 (48.3)	
Unknown	2227	375	

Grade			
Well, moderately, or poorly diff.	225 (31.3)	60 (35.5)	0.314
Undifferentiated	493 (68.7)	109 (64.5)	
Unknown	2227	461	

Primary site bone			
Yes	2065 (70.1)	354 (56.2)	0.001
No	880 (29.9)	276 (43.8)	

Primary site bone, axial^*∗*^			
Yes	371 (18.0)	15 (4.2)	0.001
No	1694 (82.0)	339 (95.8)	

Primary site bone, pelvis^*∗*^			
Yes	446 (21.6)	20 (5.7)	0.001
No	1619 (78.4)	334 (94.3)	

^*∗*^Among patients with bone primary tumors (*n*=2065 for ES; *n*=354 for PNET).

## Data Availability

The authors used the Surveillance, Epidemiology, and End Results (SEER) program, which is supported by the Surveillance Research Program (SRP) in the National Cancer Institute's (NCI) Division of Cancer Control and Population Sciences (DCCPS). This program is free of charge and accessible by anyone with an Internet connection or via DVD sent via United States mail. One may access these data readily, without cost, by visiting the SEER website at https://seer.cancer.gov/.

## Supplementary Materials

Supplementary Materials Demographic and clinical features of patients with Ewing sarcoma and PNET diagnosed in the 1990s or later (*n*=3575).
