# Supplementary material for: Comparison of Epidemiology, Clinical Features, and Outcomes of Patients with Reported Ewing Sarcoma and PNET over 40 Years Justifies Current WHO Classification and Treatment Approaches
Source: Sarcoma. 2018 Aug 8;2018:1712964. doi: 10.1155/2018/1712964 (PMC6109476; doi:10.1155/2018/1712964)
Supplement: Supplementary Materials — Demographic and clinical features of patients with Ewing sarcoma and PNET diagnosed in the 1990s or later (n=3575). [file 1712964.f1.docx]

**Supplemental Table:** Demographic and clinical features of patients with Ewing sarcoma and PNET diagnosed 1990 or later (n=3575).

|  | **Ewing Sarcoma**  **(N = 2512)** | **PNET**  **(N = 551)** | **p-value** |
| --- | --- | --- | --- |
| N | N (%) | N (%) |  |
| **Gender**  Male  Female | 1513 (60.2%)  999 (39.7%) | 295 (53.4%)  256 (46.6%) | 0.004 |
| **Age at diagnosis**  0-18 years  19+ years | 1284 (51.1%)  1228 (48.9%) | 204 (37.0%)  347 (63.0%) | <0.001 |
| **Race**  White  Black  Other*  Unknown | 2253 (90.0%)  80 (3.1%)  171 (6.9%) | 469 (85.7%)  32 (5.8%)  46 (8.4%)  4 | 0.005 |
| **Ethnicity**  Hispanic  Non-Hispanic | 528 (20.0%)  1984 (79.0%) | 50 (9.1%)  501 (90.9%) | <0.001 |
| **Metastasis at diagnosis**  Yes  No  Unknown | 306 (25.2%)  956 (75.7%)  1250 | 72 (24.4%)  223 (75.6%)  256 | 0.504 |
| **Maximum tumor dimension**  <8cm  >/=8cm  Unknown | 379 (48.1%)  409 (51.9%)  1724 | 101 (49.5%)  103 (50.5%)  347 | 0.753 |
| **Grade**  Well, moderately or poorly diff.  Undifferentiated  Unknown | 179 (29.4%)  429 (70.6%)  1904 | 52 (35.9%)  93 (64.1%)  406 | 0.134 |
| **Primary site bone**  Yes  No | 1817 (72.3%)  695 (27.6%) | 314 (57.0%)  237 (43.0%) | 0.000 |
| **Primary site bone, axial**  Yes  No | 492 (19.6%)  2020 (80.4%) | 15 (4.2%)  339 (95.8%) | 0.000 |
| **Primary site bone, pelvis**  Yes  No | 446 (21.6%)  1619 (78.4%) | 29 (5.3%)  522 (94.7%) | 0.000 |

*Includes: Asian or Pacific Islander, American Indian or Alaska Native
